# Nutrition in the prevention and treatment of endometriosis: A review

**DOI:** 10.3389/fnut.2023.1089891

**Published:** 2023-02-17

**Authors:** Neal D. Barnard, Danielle N. Holtz, Natalie Schmidt, Sinjana Kolipaka, Ellen Hata, Macy Sutton, Tatiana Znayenko-Miller, Nicholas D. Hazen, Christie Cobb, Hana Kahleova

**Affiliations:** ^1^Department of Clinical Research, Physicians Committee for Responsible Medicine, Washington, DC, United States; ^2^Adjunct Faculty, George Washington University School of Medicine and Health Sciences, Washington, DC, United States; ^3^Charles E. Schmidt College of Medicine, Florida Atlantic University, Boca Raton, FL, United States; ^4^Milken Institute School of Public Health George Washington University, Washington, DC, United States; ^5^Department of Obstetrics and Gynecology, Medstar Georgetown University Hospital, Washington, DC, United States; ^6^Department of Obstetrics and Gynecology, Baptist Health Medical Center, Little Rock, AR, United States

**Keywords:** endometriosis, nutrition, nutrients, vitamins, plant-based

## Abstract

Endometriosis is characterized by the presence of endometrial tissues outside the uterine lining, typically on the external surface of the uterus, the ovaries, fallopian tubes, abdominal wall, or intestines. The prevalence of endometriosis in North America, Australia, and Europe is ~1–5% in women of reproductive age. Treatment options for endometriosis are limited. While over-the-counter medications may be used to reduce acute pain, hormonal treatments are common and may interfere with fertility. In more severe cases, laparoscopic excision procedures and even hysterectomies are used to treat the pain associated with endometriosis. Nutritional interventions may be helpful in the prevention and treatment of endometriosis and associated pain. Reducing dietary fat and increasing dietary fiber have been shown to reduce circulating estrogen concentrations, suggesting a potential benefit for individuals with endometriosis, as it is an estrogen-dependent disease. Meat consumption is associated with greater risk of developing endometriosis. Anti-inflammatory properties of plant-based diets may benefit women with endometriosis. Additionally, seaweed holds estrogen-modulating properties that have benefitted postmenopausal women and offers potential to reduce estradiol concentrations in pre-menopausal women. Furthermore, consumption of vitamin D has been shown to reduce endometrial pain *via* increased antioxidant capacity and supplementation with vitamins C and E significantly reduced endometriosis symptoms, compared with placebo. More randomized clinical trials are needed to elucidate the role of diet in endometriosis.

## Introduction

Endometriosis is characterized by the presence of endometrial tissues outside the uterine lining, typically on the external surface of the uterus, the ovaries, fallopian tubes, abdominal wall, or intestines (1). Common symptoms include severe pelvic pain, dysmenorrhea, dyspareunia, fertility complications, fatigue, low back pain, bloating, constipation, and diarrhea. Because symptoms may be non-specific, diagnosis is often delayed (1–3).

The prevalence of endometriosis in North America, Australia, and Europe is ~1–5% in women of reproductive age, and some have suggested that the true figure is closer to 10% (4, 5). The disease leads to substantial medical costs for laparotomy, laparoscopy, oophorectomy, hysterectomy, and other procedures, estimated in 2018 at ~$10,000 annually per patient in the U.S., in addition to indirect costs related to the interference with work and other obligations (6). Surgery is often recurrent (7, 8).

A role for nutrition in endometriosis is suggested by the influence of diet on estrogenic activity and inflammatory processes. This article reviews current evidence regarding nutrition in the prevention and treatment of endometriosis.

## Pathophysiology of endometriosis

Various theories exist to explain the etiology of endometriosis (9). A widely cited hypothesis is ovarian metaplasia *via* the coelomic epithelium, leading to the formation of cysts or lesions in the peritoneal cavity (9, 10).

Also commonly accepted is the migration of endometrial tissues outward through the fallopian tubes (5). In the peritoneal cavity, endometrial tissue is associated with increased levels of prostaglandins, cytokines, and chemokines, all of which are markers of inflammation. Estradiol may be synthesized locally from cholesterol within the lesion, thus encouraging growth and inflammation amongst endometriotic tissue (11).

Endometriosis is a chronic condition that is usually developed several years after menarche. It generally wanes after menopause. The treatment options and/or nutrition interventions are the same for all age groups.

## Current treatment options

The acute pain of endometriosis often initially responds to over-the-counter non-steroidal anti-inflammatory drugs. However, these medications have limited effectiveness, as well as some adverse effects that limit their use (12).

Hormonal treatments focus on the suppression of endogenous estrogen production and include progestins, combined oral contraceptives, gonadotrophin-releasing hormone agonists and antagonists, testosterone analogs, and aromatase inhibitors (13, 14). Hormone-modulating therapies such as gonadotrophin-releasing hormones used to repress ovarian function are not recommended for patients who are pregnant or wishing to conceive (5, 15). Treatment discontinuation is common, often related to side effects, such as weight gain, nausea, headaches, vasomotor instability, decreased libido, and mood changes (16, 17).

A cross-sectional study estimated that 60% of patients had chronic pain regardless of medical treatment, along with adverse effects on quality of life (18). A 2017 systematic review found that at least 10% of patients had no pain reduction following pharmaceutical treatment and up to 60% of patients reported continued pain after treatment cessation (19).

Laparoscopic excision and ablation surgeries involve removal of pathological lesions *via* cutting and cauterization. Excision is typically preferred over ablation as it is associated with better pain outcomes (20). Differentiating pathological adhesions from healthy tissue requires a specialized knowledge, and incomplete resection may confer the need for additional surgeries (21).

A 2004 randomized single-blind control trial compared the efficacy of laparoscopic excision vs. a staging laparoscopy without excision of lesions in 39 patients (22). The patients were not aware of which procedure they had undergone. In the excision group, 80% of participants reported symptomatic improvement, compared with 32% of the non-excision group. Six months later, all participants had a follow-up laparoscopic examination. Of those who had had the initial excision procedure, biopsy-proven endometrial lesions had recurred in 43%, while 31% had no evidence of endometriosis. An additional 25% had suspicious lesions that proved biopsy negative. Among those who had had the initial non-excision procedure, the follow-up laparoscopy identified disease that was unchanged in 33%, worse in 45%, and improved in 22% (22). Subsequent studies examined the efficacy of surgery for endometriosis symptoms, but conclusions have been cautious. A 2020 Cochrane review including 14 randomized trials concluded that, “compared to diagnostic laparoscopy only, it is uncertain whether laparoscopic surgery reduces overall pain associated with minimal to severe endometriosis (23).”

Hysterectomy is effective in reducing pain and symptom recurrence in endometriosis, but is major surgery and, of course, ends fertility (24). A Swiss study found that nearly half of endometriosis patients surveyed were dissatisfied with their medical support (25). The limited efficacy, risk of complications, and low patient satisfaction with treatment options has led to interest in additional treatment options.

## Nutrition and endometriosis

Research on the relationship between nutrition and the risk of developing endometriosis or on the efficacy of nutritional treatments is far from complete (26). Nonetheless, a number of studies have raised observations that support a role for nutrition interventions.

### Dietary fat

Fat quality and quantity appear to be modulating factors for endometriosis. A 2010 prospective study found that the intake of palmitic acid (a saturated fatty acid derived mainly from meat and dairy products) and trans fat was associated with an increased risk of endometriosis, while total fat consumption did not confer the same risk (27). Although artificial trans fats have been banned in the United States, trans fats are naturally present in meat and milk derived from cows and sheep (28).

Animal fats are of particular interest as high consumption has been linked to other gynecological diseases. The NIH-AARP Diet and Health Study found that participants with the highest total fat intake had a 28% greater risk for ovarian cancer compared to those with the lowest intake; only animal-derived fats had a significant positive correlation (29). Whether animal fats similarly influence the risk of developing endometriosis remains unknown.

In contrast, certain fats may play protective roles. A 2010 study following 70,709 premenopausal women reported that those consuming the most omega-3 fatty acids were less likely to be diagnosed with endometriosis, compared with those with the lowest omega-3 consumption (27).

Because estrogens are key in the pathogenesis of endometriosis, dietary factors that modulate estrogen activity may be clinically important. Reducing dietary fat and increasing dietary fiber has been shown to reduce circulating estrogen concentrations by roughly 10 to 25 percent (20, 30, 31). The same phenomenon has been identified in postmenopausal women (32). In addition, some have suggested that estrogens occurring in animal products, particularly dairy and meat products, may have biological effects when ingested (33).

### Meat intake

Two prospective studies demonstrated significant correlations between red meat consumption (both unprocessed and processed) and the risk of developing endometriosis (34, 35). The Nurses' Health Study II showed that women consuming more than two servings of red meat per day had a 56% greater risk of endometriosis, compared with those consuming less than one serving of red meat per week (RR 1.56, 95% CI 1.22, 1.99) (35). Increased poultry intake was also associated with higher risk of endometriosis (RR 1.14, 95% CI 0.88, 1.49), although risk estimates for specific intake categories were not significant. Increased risk associated with fish and shellfish intake was no longer apparent after adjustment for race, parity, body mass index, age at menarche, menstrual cycle length, pelvic or breast exam in the past year, and energy intake. An Italian study reported increased odds of developing endometriosis associated with a higher intake of beef, other red meat, and ham (34). Red meat consumption may be associated with higher levels of estradiol and estrone sulfate, and thereby with higher concentrations of steroids, inflammation, and the development of endometriosis (36). Furthermore, red meat consumption may promote expression of pro-inflammatory markers, which appear to be implicated in endometriosis pathogenesis and progression (37). A summary of the association of different kinds of meat with the risk of endometriosis is shown in Table 1.

**Table 1:** Dietary factors under investigation and their role in endometriosis.

**Dietary factor**	**Biological role in endometriosis**
**Animal products**	
Red meat, unprocessed	Associated with an increased risk of edometriosis (34, 35)
Red meat, processed	Associated with an increased risk of edometriosis (34, 35)
Dairy products	Contain estradiol and palmitic acid, associated with an increased risk of endometriosis (27, 33)
Dietary fat	Increases circulating estrogen concentrations (20, 30, 31, 33)
**Plant-based components**	
Dietary fiber	Reduces circulating estrogen concentrations (20, 30, 31)
Seaweed	Estrogen-modulating activity (44)
Polyphenols	Anti-inflammatory (39)
Vitamin D	Anti-inflammatory activity (47)
Vitamins C and E	Antioxidant activity, reduce endometriosis symptoms (50, 51)

### Plant-based diets

Because of the role of inflammation in endometriosis, and the fact that reducing dietary fat and increasing dietary fiber reduce circulating estrogen concentrations, the effects of plant-based diets on inflammation have been of clinical interest. An 8-week study of 100 participants with coronary artery disease published by the American Heart Association found that those following a strictly vegan diet experienced less inflammation than those following a diet including animal products (38). A 2021 literature review confirmed the anti-inflammatory properties of a plant-based diet (39).

In a randomized crossover trial in women with dysmenorrhea, a low-fat vegan diet was shown to increase plasma concentrations of sex-hormone binding globulin, which, in turn, would be expected to reduce estrogen activity. The diet also reduced severity and duration of pain and moderated premenstrual symptoms (40).

Observations from the Nurses' Health Study II, including 81,961 premenopausal women, suggested that low-Glycemic Index foods are associated with reduced risk of endometriosis (41). Increased fruit fiber has been associated with a reduced risk of endometriosis in two independent studies (34, 42).

Plant-based foods contain increased amounts of polyphenols when compared to an omnivorous diet, which, when metabolized into bioactive compounds, can reduce inflammation (39). When comparing a plant-based diet to an omnivorous diet, the gut microbiome contains a larger number of anti-inflammatory compounds (43). As noted above, a crossover trial using a low-fat plant-based (vegan) vs. a placebo demonstrated significant benefits of the dietary intervention in dysmenorrhea; application to endometriosis awaits further research.

### Seaweed

Seaweed may have estrogen-modulating properties. A double-blind trial testing the effects of seaweed in healthy postmenopausal women found an inverse dose-response relationship between seaweed consumption and serum estradiol concentrations, i.e., the higher the seaweed consumption, the lower the estradiol concentrations (44), which may partly explain its protective effects for breast cancer and lower postmenopausal breast cancer incidence and mortality in Japan (39). Seaweed may also reduce estradiol concentrations in pre-menopausal women. A case study in three pre-menopausal women reported that intake of bladderwrack, an edible brown kelp, significantly increased the length of menstrual cycle and reduced estradiol levels (45). These studies suggest potential benefits of seaweed consumption on estradiol concentrations, but effects on endometriosis await further research.

### Vitamin D

Vitamin D may play a role in the prevention and treatment of endometriosis. A 2020 meta-analysis found that low vitamin D levels were associated with increased risk of endometriosis diagnosis and increased severity of symptoms (46). In a randomized, placebo-controlled trial in women with endometriosis, 50,000 IU vitamin D every 2 weeks for 12 weeks reduced pelvic pain by 1.12 points on a self-reported scale. It was also associated with reduced high-sensitivity C-reactive protein and increased total antioxidant capacity, suggesting that an antioxidant and anti-inflammatory effect may be involved (47).

### Antioxidants

Free radicals are generated through physiological processes and exposure to environmental factors and cause oxidative stress, which often results in cell damage or death (48), contributing to a wide range of pathologies (49). Conversely, antioxidants are molecules that combat free radicals and can be endogenously synthesized or exogenously consumed. Because of the role of inflammation in endometriosis, research studies have addressed the potential role of antioxidant intake. A 2009 study from Mexico reported that women with endometriosis had a 30% lower intake of vitamin C and consumed 40% less vitamin E, compared with women who did not have endometriosis. Following 3 months on a high-antioxidant diet, women with endometriosis displayed higher peripheral concentrations of supplemented vitamins (50). A later randomized control trial found that supplementation with vitamins C and E significantly reduced endometriosis symptoms, compared with placebo (51).

## Conclusions

The pathophysiology of endometriosis involves the actions of estrogens and inflammatory processes. Evidence suggests that dietary factors have important effects in both of these domains. Consumption of trans fats, palmitic acid, and red meat is associated with increased risk of endometriosis, while factors in plant-based foods, particularly fiber and antioxidants, and vitamin D may have helpful effects for prevention and treatment. Further investigations, particularly randomized clinical trials, will be helpful in elucidating the role of diet in endometriosis.

## Author contributions

All authors contributed to the manuscript and approved its final version.
